# Three different indications for left ventricular unloading in one patient with severe heart failure

**DOI:** 10.1016/j.ahjo.2026.100716

**Published:** 2026-01-09

**Authors:** Thomas Gausepohl, Ulrike Flierl, Vera Garcheva, Marcel Ricklefs, Bernd Schwedhelm, Johann Bauersachs, Tobias J. Pfeffer, Andreas Schäfer

**Affiliations:** a Department of Cardiology and Angiology, Medical School Hannover, Germany; b Department of Cardiology, Klinikum Region Hannover Siloah, Germany

**Keywords:** Impella, Mechanical circulatory support, Heart failure

## Abstract

**Background:**

The percutaneous microaxial flow pump (mAFP) is an established mechanical circulatory support (MCS) device for cardiogenic shock (CS) and can also stabilize hemodynamics during cardiac and noncardiac procedures. This case series describes the safety and feasibility of repeated mAFP use for three indications in a single patient.

**Methods:**

We retrospectively reviewed three consecutive mAFP deployments in one patient enrolled in the Hannover-Cardiac-Unloading-REgistry between October 2021 and August 2023. Indications included acute myocardial infarction–related CS (AMI-CS), ventricular tachycardia (VT) ablation one year later, and elective thyroidectomy for amiodarone-induced hyperthyroidism another year later.

**Results:**

A 62-year-old patient with ischemic cardiomyopathy (initial LVEF 34%) achieved hemodynamic stabilisation during AMI-CS, successful protected VT ablation without compromise, and stable perioperative support during thyroidectomy. No major adverse events occurred.

**Conclusions:**

Repeated mAFP use for AMI-CS, VT ablation, and high-risk surgery was feasible and safe, supporting its versatility in complex clinical care.

## 1 Introduction

The percutaneous transvalvular microaxial flow pump (mAFP) is an effective tool in order to support the left ventricle's (LV) haemodynamic capacity by pumping blood directly from the LV into the ascending aorta [1]. In the randomized, controlled DanGer-Shock trial mAFP in addition to standard care reduced mortality in cardiogenic shock (CS) related to myocardial infarction (AMI-CS) [2]. Furthermore, succesfull mAFP can be used to maintain hemodynamic stabilisation in patients with heart failure (HF) undergoing procedures associated with hemodynamic stress like ablation of ventricular tachycardia (VT) or during emergent and nonemergent noncardiac surgery (NCS) [3], [4].

The aim of this case series is to describe three cases of mAFP use for three different indications in one single patient at three different points of time with special regard to safety and feasibility of repeated mAFP derived circulatory support.

## 2 Methods

### 2.1 Study design and participants

The cases were enrolled in the prospective and observational Hannover-Cardiac-Unloading-REgistry (HACURE). The shown data accords with the Declaration of Helsinki and was approved by the Hannover Medical School ethics committee (#3566–2017).

The cases are dated between october 2021 and august 2023.

### 2.2 Patient treatment

Implantation of mAFPs was performed during AMI-CS (#1) and NCS (#3) at the department of Cardiology and Angiology at Hannover Medical School while protected VT-ablation (#2) was performed at the department of Cardiology at Klinikum Region Hannover Siloah. CS is defined in HACURE when LV function is depressed and systolic blood pressure is <90 mmHg or inotropes/vasopressors are required to maintain systolic blood pressure > 90 mmHg, and criteria of end-organ hypoperfusion are observed after preload correction (e.g. elevated arterial lactate [>2 mmol/l] reflecting Society of Cardiovascular Angiography & Intervention (SCAI) class C or worse CS) [5].

Ultrasound guided femoral access was used for implantation of Impella-CP in all 3 cases and echocardiographic control for device position was regularly performed (at least every eight hours).

Unfractionated heparin was used for anticoagulation during MCS (activated clotting time of 160–180 s). HF treatment was performed according to the current European Society of Cardiology guidelines for treatment of acute HF, in case of CS extended with the local standard operating procedure for CS [6].

After demission, repeated VT-episodes emerged: need for mAFP-protected VT-ablation and later NCS was assessed by interdisciplinary judgement in the HeartTeam (including an electrophysiologist (case 2) or surgeon (case 3), endocrinologist (case 3), anesthetist (case 3) and interventional cardiologist and intensivist) balancing risk for omitting the intervention/operation to the risk of intervention/operation and anesthesia without MCS in the context of severe HF.

### 2.3 Statistical analysis

Numbers are presented as n.

## 3 Results

### 3.1 Patient characterstics

At time of initial presentation, the patient was 62 years old and had a medical history including coronary artery disease (CAD) and ischemic HF (tbl. 1). Regarding CAD, the patient had received multiple percutaneous coronary interventions (PCI) (*n* = 4) before and was about to be scheduled for an ICD implantation due to worsening HF (LVEF = 35%). During diagnostic work-up of the LVEF deterioration, angiography was performed at a referring hospital and a re-stenosis was detected in the right coronary artery (RCA) following multiple previous PCIs with dual-layer stenting. Another PCI was performed including stenting to resolve coronary ischemia resulting in triple-layer stenting.

### 3.2 mAFP in AMI-CS

**Fig. 1:**
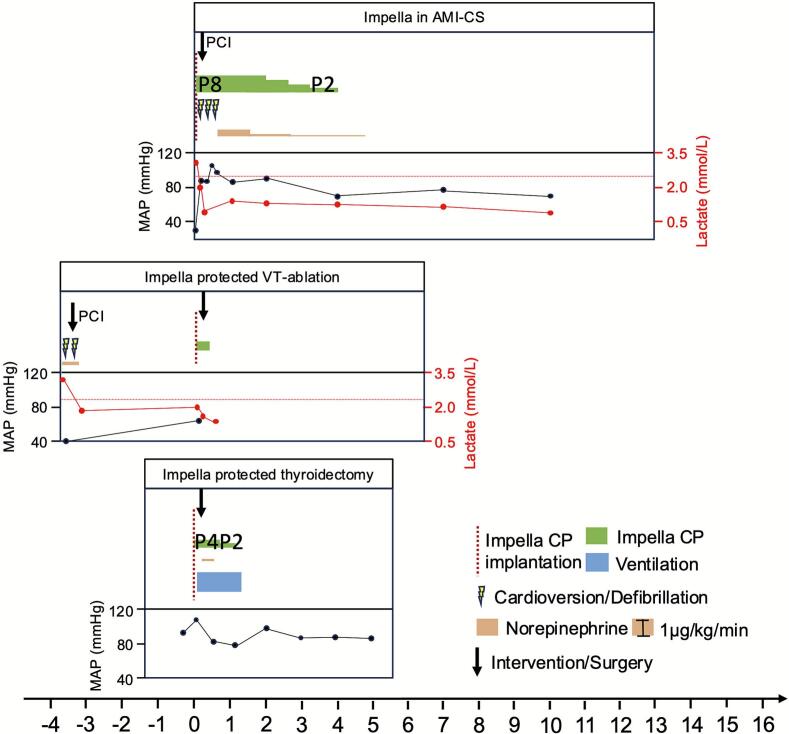
Treatment course of Impella use in the settings of acute myocardial infarction related cardiogenic shock (AMI-CS), protected ablation of ventricular tachycardias (VT) and protected thyroidectomy. MAP = mean arterial pressure.

Four days after the PCI of the RCA at the referring hospital, the patient was admitted to our centre with AMI-CS triggered by stent-thrombosis. mAFP was established prior to PCI, then successful PCI of the RCA was performed (TIMI III flow). The maximum CK amounted to 2050 U/l. The patients rapidly had hemodynamic stabilisation after PCI and mAFP insertion and pulmonary arterial pressure and central venous pressure decreased by more than half within the first 24 h to within the normal range (Fig. 1). mAFP was used for 95 h and the patient was discharged from intensive care after 11 days without cognitive impairement. During rehabilitation after AMI, the patient had repetitive VT's, received an ICD in a different hospital and was initiated on amiodarone treatment.

### 3.3 Protected VT-ablation

About one year later, the patient was admitted to another hospital due to electric storm and several shock deliveries from the ICD. The patient was stabilized and transferred for VT ablation. Due to the worsened LVEF in this patient with extensive HF, VT-ablation was conducted as protected ablation, which was successfully performed on mAFP. Haemodynamics remained stable throughout the procedure and the mAFP was rapidly removed after 240 min.

### 3.4 mAFP protected non-cardiac surgery

Following the initial VT-ablation, however, the patient continued to suffer from symptomatic VTs. During treatment with amiodarone, VT-burden was reduced, but the patient developed amiodarone-induced hyperthyroidism, both potentially accelerating arrhythmia burden and prohibiting further amiodarone treatment. Following discontinuation of amiodarone, VT-burden increased, highlighting the need for reimplementation of amiodarone, which would only be possible after eliminating hyperthyroidism by surgical thyroidectomy. However, the procedural risk was estimated as extremely high based on the further declined LVEF of 22%. After interdisciplinary risk assessment regarding pre-existing severe HF a potential hemodynamic protection during thyroidectomy using an mAFP was discussed. At the morning of surgery, the patient was first taken to the cath-lab, mAFP was implanted percutaneously in local anesthesia with mild analgosedation (2 mg midazolam). Afterwards, the awake patient was directly transferred to the operating-room accompanied by an interventional cardiologist, who was co-specialized in intensive care. Induction of anesthesia as well as the surgical procedure were carried out without any hemodynamic deterioration, the patient remained fully stable, was kept in general anesthesia over night to minimize the risk of hemodynamic compromise in case of surgical re-do, and the mAFP was explanted 24 h after surgery in the cath-lab using a large-bore post-closure device. After arriving back on the cardiology ICU, the patient was safely extubated and transferred to a peripheral cardiology ward the day after.

### 3.5 Adverse events

As stated in Table 1, repetitive mAFP deployment neither induced any significant bleeding nor haemolysis and the neurological outcome was positive. Besides a mild acute kidney injury no adverse events could be observed.

**Table 1:** Clinical characteristics.

Baseline characteristics	
BMI (kg/m^2^)	25
Height (cm)	180
Weight (kg)	81
Smoking history	+ (terminated)
Chronic obstructive pulmonary disease	−
Arterial hypertension	+
Diabetes mellitus	+
Pre-existing heart failure	+ (ischemic cardiomyopathy)
Chronic renal failure	+
Liver cirrhosis	−


	AMI-CS	Imp. prot. VT-abl.	Imp. prot. surgery
Age (years)	62	63	63
Transferred from secondary hospital	No	Yes	No
Shock Score SCAI	D	D	−
Shock to Impella	<6 h	>24 h	−
Duration Impella	95 h	4 h	24 h
ECMO	−	−	−
Duration Ventilation	−	−	25 h
Duration hospital stay (days)	13	10	7
Duration ICU stay (days)	11	3	3
CK (max) U/l (ULN 171)	2050	1155	−
cTroponin T ng/l (ULN 14)	3822	1700	−
LVEF (%)	34	23	22
LVEDD (cm)	5.9	6.6	6.8

Outcome			
Haemolysis	None	None	None
TIMI Bleeding	Minimal	None	None
AKI	None	Stage 1	None
CPC Score	1	1	1

Data are stated as n. AKI = acute kidney injury, CK = creatine kinase, CPC = cerebral performance category, ECMO = extracorporeal membrane oxygenation, ICU = intensive care unit, LVEF: Left ventricular ejection fraction, LVEDD: Left ventricular end-diastolic dimension, SCAI = Society for Cardiovascular Angiography and Interventions, ULN = upper limit of normal, TIMI = Thrombolysis in Myocardial Infarction bleeding Score.

## 4 Discussion

Circulatory support using mAFPs improves haemodynamic stabilisation and reduces mortality in AMI-CS as well as maintains haemodynamic stabilisation in the setting of VT-ablation and NCS [2], [3], [4]. With increasing survival rates in AMI-CS, more HF patients will result a growing population of patients that will inherit higher intraoperative risk and elevated morbidity/need for interventional/surgical treatment in case of other NCS [2]. In this case series, survival of AMI was followed by ventricular tachycardias as consequence of the underlying ischemic heart disease as well as hyperthyroidism induced by treatment with the anti-arrhythmic drug amiodarone. HF patients display increased perioperative risk of hemodynamic decompensation [7]. The underlying challenges for the weakened heart are reduction in sympathetic tone, myocardial depression and coping complications such as bleeding events under surgery or tachycardias during ablation procedures [8]. Here, mAFP served as a fallback level and enabled reliable cardiac output generation in case of perioperative complications and unloaded the LV pressure thereby optimizing myocardial perfusion and targeting myocardial depression [1]. The stable haemodynamics under mAFP and rapid clearance of lactate in all three cases of mAFP use support this notion (Fig. 1).

While effects of long duration cannulation and its infectious and neurological side effects have been intensively studied, data regarding feasability of repetetive cannulation are rare [9], [10].

Hereby we showed that repetitive use of mAFP during AMI-CS followed by protected ventricular ablation and NCS was safe and resulted in a good outcome.

### 4.1 Limitations

Besides invasive blood pressure values, more extensive invasive hemodynamic data during mAFP protected VT-ablation and non-cardiac surgery are lacking.

## CRediT authorship contribution statement

**Thomas Gausepohl:** Writing – original draft, Investigation, Formal analysis, Data curation. **Ulrike Flierl:** Writing – review & editing, Investigation, Data curation. **Vera Garcheva:** Writing – review & editing, Formal analysis. **Marcel Ricklefs:** Writing – review & editing, Data curation. **Bernd Schwedhelm:** Writing – review & editing, Formal analysis. **Johann Bauersachs:** Writing – review & editing, Formal analysis, Conceptualization. **Tobias J. Pfeffer:** Writing – original draft, Formal analysis, Data curation, Conceptualization. **Andreas Schäfer:** Writing – review & editing, Writing – original draft, Formal analysis, Data curation, Conceptualization.

## Ethical statement

Hereby the authors declare, that the shown data accords with the Declaration of Helsinki and was approved by the Hannover Medical School ethics committee (#3566-2017).

## Sources of funding

HACURE was supported by research funds from Abiomed (Danvers, MA, USA). The funders had no role in study design, data collection and analysis, decision to publish, or preparation of the manuscript.

## Declaration of competing interest

AS received lecture fees and honoraria from Abiomed, Amgen, AOP, Boehringer Ingelheim, BMS, Daiichi-Sankyo, Eli Lilly, Novartis, Pfizer, ZOLL as well as research support by Abiomed and Daiichi-Sankyo. JB received lecture fees and honoraria from Novartis, Abbott, Bayer, Pfizer, Boehringer Ingelheim, AstraZeneca, Cardior, CVRx, BMS, Amgen, Edwards, Roche, Zoll not related to this article; and research support for the department from Zoll, CVRx, Abiomed, Norgine, Roche, not related to this article. TJP received honoraria for lectures/consulting from AstraZeneca not related to this article. The other authors declare that they have no conflict of interest, particularly not related to the current manuscript.
